# Comparison of Gaseous and Water-Based Medium-Expansion Foam Depopulation Methods in Cull Sows

**DOI:** 10.3390/ani11113179

**Published:** 2021-11-07

**Authors:** Joshua N. Lorbach, Magnus R. Campler, Brad Youngblood, Morgan B. Farnell, Tariku J. Beyene, Justin Kieffer, Steven J. Moeller, Andréia G. Arruda, Andrew S. Bowman

**Affiliations:** 1 Department of Veterinary Preventive Medicine, College of Veterinary Medicine, The Ohio State University, Columbus, OH 43215, USA; lorbach.5@osu.edu (J.N.L.); campler.1@osu.edu (M.R.C.); youngblood.25@osu.edu (B.Y.); arruda.13@osu.edu (A.G.A.); 2 Department of Poultry Science, College of Agriculture & Life Sciences, Texas A & M University, College Station, TX 77843, USA; mfarnell@tamu.edu; 3 Center for Surgical Outcomes Research, The Research Institute at Nationwide Children’s Hospital, Columbus, OH 43215, USA; Tariku.Beyene@nationwidechildrens.org; 4 Department of Animal Sciences, College of Food, Agricultural, and Environmental Sciences, The Ohio State University, Columbus, OH 43215, USA; kieffer.22@osu.edu (J.K.); moeller.29@gmail.com (S.J.M.)

**Keywords:** swine, depopulation, aspirated foam, disease

## Abstract

**Simple Summary:**

In the face of a swine health crisis, emerging zoonotic diseases or environmental catastrophe, the mass depopulation of swine may be required to prevent the additional spread of disease and to minimize animal pain or suffering. Due to the increasing risk of global disease outbreaks, the U.S. swine industry needs feasible guidelines in place in preparation for such events. Current American Veterinary Medical Association (AVMA) approved swine depopulation methods can be difficult to implement under field conditions. Emergency depopulation using inhalants such as carbon dioxide (CO_2_) and nitrogen gas (N_2_) or the use of aspirated foam agents have been approved and conducted in poultry in the US, but are not approved for use in other livestock. Our findings, using cull sows, demonstrate that although CO_2_, N_2_ and aspirated foam combinations successfully killed all the animals, CO_2_ and aspirated foam did so in the shortest timeframe. In addition, the use of aspirated foam was as effective as CO_2_ for sow depopulation while having potential operational advantages, such as no use of lethal gases and reduced risk of associated equipment failure.

**Abstract:**

The U.S. swine industry is currently inadequately prepared to counteract the increasing threat of high-consequence diseases. Although approved and preferred depopulation guidelines exist, ventilation shutdown (VSD+) is currently the only method being deployed during a state of emergency to depopulate large swine populations. However, the permitted use of VSD+ during constrained circumstances has been criticized due to raised swine welfare concerns. The objective of this study was to investigate the effectiveness of carbon dioxide gas (CO_2_), nitrogen gas (N_2_), compressed air foam (CAF), compressed nitrogen foam (CAF-N_2_) and aspirated foam (AF) during a 15-min dwell time on adult swine in an emergency depopulation situation. A small-scale trial using 12 sows per depopulation method showed the highest efficiency to induce cessation of movement for AF and CO_2_ (186.0 ± 48 vs. 202.0 ± 41, s ± SD). The ease of implementation and safety favored AF for further investigation. A large-scale field study using AF to depopulate 134 sows in modified rendering trailers showed a mean fill time of 103.8 s (SD: 5.0 s) and cessation of movement of 128.0 s (SD: 18.6 s) post filling. All sows were confirmed dead post-treatment for both trials. The implementation of AF in modified rendering trailers may allow for a safe and reliable method that allows for the expedient and mobile depopulation of both small and large numbers of sows during an emergency.

## 1. Introduction

Modern swine production coupled with the increased global movement of people and animals increases the chance of the introduction and spread of pathogens. Disease outbreaks on swine farms negatively impact animal health and welfare, public health [1], the food supply chain and the economy [2,3]. They may also negatively affect public perception of swine production and the public’s view on government responses in dealing with outbreaks [4,5]. As an example, African Swine Fever (ASF) is a severe viral disease affecting domestic and feral swine, for which there is currently no commercially available vaccine [6]. Although the U.S. swine herd is free of ASF, ongoing transmission throughout China, parts of Europe [7] and more recently, the Western Hemisphere [8,9], show that pathogens can travel with the global transportation network of feed, replacement animals, pork products and equipment [6]. These findings indicate that an outbreak occurring on the other side of the world can suddenly appear and spread locally or regionally with no or little warning. Recent outbreaks highlight the importance of having readily available contingency plans in place for the U.S. swine industry.

Even though there are informative guidelines developed by the American Veterinary Medical Association (AVMA) on swine depopulation and euthanasia, applying recommended measures to large populations of swine under field conditions is a challenge [9]. Modern swine facilities house large numbers of swine and commonly comprise multiple buildings, rooms and types of environmental conditions. To ensure a death as humane and rapid as possible while simultaneously being attentive to human safety and mental challenges, a defined set of operational parameters is essential [10,11,12,13]. In brief, these parameters are: (1) caretakers should have minimal physical interaction with animals to minimize injury or spread of disease, (2) the depopulation methodology should pose little to no risk to the caretakers during a normal performance and (3) any subdivision of animals, due to partial depopulation or different depopulation methodologies and requirements should be moved quickly and safely to a designated depopulation area [14]. Therefore, as recommended by the AMVA, developing and testing a depopulation plan before an incident occurs becomes imperative. For the animals, the goal of best practice depopulation systems is to minimize or eliminate animal anxiety, pain and distress before the loss of consciousness. Thus, when evaluating alternative systems, both the handling/restraint processes and induction of unconsciousness should be considered. AVMA’s current preferred depopulation methods include physical methods (non-penetrating captive bolt, penetrating captive bolt, electrocution, manual blunt force trauma and movement to slaughter), one inhaled method (carbon dioxide) and one non-inhaled method (anesthetic overdose) [9,10]. Recently, ventilation shutdown (VSD+) has been used for the emergency depopulation of large swine populations in the U.S. but is currently listed in the AVMA depopulation guidelines to be used solely under ‘constrained circumstances’ as a last resort [10]. However, many of these methods are not good candidates for the mass depopulation of large farms because they are either too time-consuming (delivered at the individual level), could represent a high risk of disease dissemination for other swine populations (e.g., movement to slaughter), may negatively impact swine welfare during the implementation or pose an additional danger to the caretaker [12]. Carbon dioxide and other inhalation methods are the most promising for larger-scale depopulation considering they would allow for multiple animals to be terminated simultaneously. However, inhalation methods such as CO_2_ and N_2_ do pose welfare concerns due to their inherent aversiveness when inhaled [14]. To date, inhalation methods for depopulation under field conditions have undergone only limited testing in swine and results have not yet provided enough guidelines for execution under emergency situations [14,15,16,17]. The National Animal Health Emergency Management System Guidelines (NAHEMS) on mass depopulation and euthanasia acknowledges that the currently approved recommended procedures may need to be adapted for field conditions [18].

Under field conditions, particularly in the presence of late finishing or large breeding animals, the ideal depopulation scenario will have animals move under their own power out of the facility prior to termination, although this may cause temporary distress or aggression between unfamiliar pigs even during limited commingling [19,20]. From a practical perspective, removing individual large animal carcasses from housing facilities is highly labor-intensive and ergonomically challenging. The manual removal of carcasses would also increase the potential for spreading infectious agents due to a greater amount of foot traffic involved with removal efforts. Delays in the timely decontamination of affected properties would also occur due to the prolonged presence of contaminated carcasses. In general, livestock carcasses being disposed of during a foreign animal disease outbreak response requires transportation/movement to a disposal site (landfill, off-site containment site or on-site disposal area) and regardless of depopulation method, would have to be transported in sealed containers to prevent the release of the infectious agent in transit. Trailers currently used for the transport of non-diseased carcasses to rendering facilities may meet these criteria. Therefore, this team began exploring the potential of depopulating groups of mature swine in a modified rendering trailer.

The objective of the current project was to investigate the effectiveness of several depopulation methods, including carbon dioxide gas, nitrogen gas, compressed air foam, compressed nitrogen foam and aspirated foam to facilitate the depopulation of sow herds in the face of an emergency.

## 2. Materials and Methods

### 2.1. Ethics and Institutional Oversight

This study was conducted according to animal use protocol 2020A00000036, which was approved by the Institutional Animal Care and Use Committee at The Ohio State University. A penetrating captive bolt device was available if a supplemental method of euthanasia was necessary at the time of assessing the consciousness of the sows, post depopulation method completion and when it was safe for operators to enter.

### 2.2. Animal Subjects

A total of 194 healthy cull sows (approximate weight: 200 to 275 kg) were purchased across multiple cohorts for small-scale and field trial experiments. The sows originated from multiple farms and delivery of animal cohorts at the research site was coordinated to permit immediate use in experiments and, as such, individual weights were not obtained prior to trial inclusion. Each trial cohort (small scale or field trial) arrived by coordinated transport trailer scheduled to arrive close to the trial start on the day of each trial. Sows chosen to be fitted with heart rate loggers or accelerometers were immediately off-loaded, while the remainder of the sows were continued to be housed within the transport trailer until the start of their trial replicate. All sows had access to feed and water in the transport trailer.

### 2.3. Small-Scale Trial Experiments

A standard, commercially available roll-off dumpster was used for the small-scale trials. Based on flow rate capacity of the gas-generating equipment, a custom reinforced wooden wall was constructed within the 9.8-m^3^ dumpster to reduce the functional volume to 4.0 m^3^ (2.21 m × 1.48 m × 1.22 m; length, width and height), accommodating industry-standard space requirements of sows during transport of at least 0.6–0.8 m^2^/sow for the sows used in the trial [21]. A top lid for the dumpster was constructed using a wood frame lined with clear poly sheeting and was placed to fully enclose the space during the application of treatments. Gaps where the swinging dumpster door met the walls and floor were eliminated using rubber weather seal. For each of the five treatments, three complete test runs without sows were conducted to ensure no leaks were present at the time of live testing.

For the small-scale trial, sixty sows were assigned by convenience (based on their placement at the trailer) to one of five treatments: carbon dioxide gas (CO_2_), nitrogen gas (N_2_), compressed air foam (CAF), compressed nitrogen foam (CAF-N_2_) and aspirated foam (AF). Each treatment was used to terminate 12 sows (six anesthetized and six conscious, in two replicates of three animals per treatment group), always starting with the anesthetized sows and moving to conscious animals for each treatment. As such, groups of three sows were walked into the empty dumpster and represented a replicate. For general anesthesia, a reconstituted mixture of telazol (50 mg tiletamine, 50 mg zolazepam powder), 2.5 mL xylazine (250 mg total) and 2.5 mL ketamine (250 mg total) was drawn at a 1 mL/27.2 kg estimated bodyweight dosage per sow. Sows in anesthetized treatment groups received a single intramuscular injection in the hind end (semimembranosus, semitendinosus or gluteal muscles) of the mentioned anesthetic combination following loading into the dumpster. The injection was concurrently administered to all sows in a treatment group and all sows were in a surgical plane of anesthesia at the time of foam or gas administration. Surgical anesthetic plane was confirmed for each animal by lateral recumbency, lack of response to noxious stimulus and lack of corneal reflex.

Individual sows in the anesthetized groups were fitted with one superficial cutaneous electrocardiography (ECG) logger—CardeaSolo (Cardiac Insight, Bellevue, WA, USA)—upon loading into the dumpster. For the superficial logger, the skin along the left lateral thorax just caudal to the elbow (approximately the 5/6th intercostal space) was clipped and shaved to remove hair, wiped with alcohol-soaked gauze and lightly exfoliated with an abrasive pad. An adhesive pad that was part of the device kit was applied to the exfoliated skin to secure the device in place before activation. Individual sows in the conscious groups were fitted with one subcutaneous logger, DST-Centi-HRT (Star-Oddi, Gardabaer, Iceland). This modification was done because the superficial loggers presented issues with adhering to the skin. Subcutaneous loggers were placed caudal to the left or right triceps by creating a 2.54-cm incision and blunt dissection to create a pocket for the device placement. The incision was closed using standard 35 W (35 mm) surgical staples. For local anesthesia in non-sedated sows, a 2% lidocaine buffered with 8.4% sodium bicarbonate in a 9:1 ratio (9 mL lidocaine, 1 mL sodium bicarbonate) was used prior to logger placement. The loggers were placed approximately immediately before treatment, and a recovery period was not specifically included given the nature of the study. Lastly, sows in conscious groups had accelerometers (HOBO Pendant G, Onset Computer Corporation, Bourne, MA, USA) secured to a distal forelimb with an elastic wrap to monitor movement and orientation. All logger functionality was tested on sows prior to the commencement of the depopulation study. However, foam submersion for each treatment type of the loggers was not conducted prior to the study start due to delivery delays from the manufacturer, resulting in a higher failure rate than expected due to the moist foam environment.

#### 2.3.1. Gas production and Application

Previously described methods for poultry depopulation were modified for this study [22,23]. Briefly, high-pressure vapor gas liquid (VGL) containers delivered either liquid CO_2_ or liquid N_2_ to a vaporizer (Thermax Inc., North Dartmouth, MA, USA) set at 65 °C. Gases flowed from the vaporizer at maximal output through in-line mass flow controllers (Alicat, Tucson, AZ, USA) and into a 947 L bulk tank. Vaporizer and mass flow controller power were supplied by a 25 kVA, 480-volt diesel engine generator (Multiquip Inc., Carson, CA, USA). The bulk tank was filled to 827.4 kpa with 100% CO_2_ gas or N_2_ gas before initiating gas delivery to the dumpster via 15 m length of fire hose. Oxygen and CO_2_ levels within the dumpster were monitored with a CO_2_ m (GasLab Pro CM-1000 Series, Ormond Beach, FL, USA). Gaseous CO_2_ or N_2_ was delivered to the dumpster to create a non-viable environment at an oxygen level at or below 5%. Once the desired oxygen level was reached, gas delivery continued for seven minutes at a rate equal to vaporizer output (59–76 m^3^/h).

#### 2.3.2. Foam Production and Application

During small-scale experiments, AF, CAF and CAF-N_2_ foam were produced using a compressed air foam system (CAFS; Rowe CAFS LLC, Hope, AR, USA). The system consisted of a 1982 L/m rotary screw air compressor (Vanair Inc., Michigan City, IN, USA), 29.42 kW (40 HP) gasoline engine (Kohler, Kohler, WI, USA), 567 L/m centrifugal water pump (Hale Products, Inc., Ocala, FL, USA) and foam proportioning unit (0.1–10%) (FoamPro, Kingston, NY, USA). The CAF unit consisted of a water manifold supplied by a 1136 L water tank and separate air manifold supplied by the air compressor (air) or vaporizer (gaseous N_2_) that fed into a mixing chamber. The foam proportioning unit was used to inject PHOS-CHEK WD881 Class A foam concentrate (Perimeter Solutions, Rancho Cucamonga, CA, USA) into the water manifold. The foam–water solution (1–2%) was agitated with gas in the mixing chamber of the CAFS unit to produce CAF (air) or CAF-N_2_ (nitrogen gas). Resulting CAF or CAF-N_2_ foams were transported from the unit through 15–30 m length fire hose (3.8 cm diameter) connected to a distal 6.0 m length suction hose (6.4 cm diameter). In the case of CAF-N_2_, 100% N_2_ gas was mixed with the foam–water solution in a mixing chamber to make the foam. For AF production, foam–water solution was transported through 3.8-cm diameter fire hose to an aspirated foam nozzle (AG-1, Spumifer American, Ridgefield Park, NJ, USA), bypassing agitation in the CAFS mixing chamber. Desired consistency and thickness of foams were achieved by adjusting the flow of aqueous foam solution. Analyses of foam properties were not conducted; therefore, variation in expansion ratio and density within and between foam treatments is possible. For AF, CAF and CAF-N_2_ treatments, the dumpster was filled with foam until it reached a depth of 122 cm (the top of the dumpster). No top-ups were required. Following filling, the lid was closed and the dumpster was not disturbed for 15 min, after which the foam was evacuated with a leaf blower and death of each sow was confirmed based on lack of heartbeat, spontaneous breathing, corneal reflex and response to noxious stimulus. Carcasses were removed from the dumpster with a tractor. Postmortem examination of large airways was performed for CAF, CAF N_2_ and AF treatment groups immediately after removal from the dumpster. Care was taken not to tip the animals vertically during removal, to aid in accurate determination of foam location within the trachea post-mortem.

### 2.4. Field Trial Experiments

Using data and findings from small-scale studies, AF was selected as the preferred method for follow-up experimentation in large group depopulation. The main reason behind this decision was the lack of peer-reviewed information on this method as applied to the swine species. In addition, AF performed well in regard to time to cessation of movement (COM) and fill-time, making it a good contender to investigate further. The field trial experiments were completed over a period of three days, using a total of 134 cull sows (n = 44 or 45 per day) originating from a single commercial farm. The sows were transported to the research site daily on a single trailer in which they were penned within three compartments and provided wood shaving bedding at recommended levels. A 45-m^3^ (dimensions 12.2 m × 1.5 m × 2.24 m; length, width and height) rendering trailer was custom modified to allow loading of sows through the rear swing door of the trailer. On each day, 44–45 cull sows were loaded directly into the rendering trailer (stocking density 0.8–1.0 m^2^/sow). Prior to the loading process, ten sows in each group were briefly restrained in the trailer for local anesthesia and subcutaneous placement of DST-Centi-HRT data loggers (Star Oddi) caudal to the left or right triceps. Subcutaneous loggers were placed caudal to the left or right triceps by creating a 2.54 cm incision and blunt dissection to create a pocket for the device placement. The incision was closed using standard 35 W (35 mm) surgical staples. For local anesthesia, 2% lidocaine buffered with 8.4% sodium bicarbonate in a 9:1 ratio (9 mL lidocaine, 1 mL sodium bicarbonate) was used prior to logger placement. Five sows were fitted with devices capable of logging movement activity (DST-Centi-HRT ACT) and five sows were fitted devices that did not log movement activity (DST-Centi-HRT) and, as such, they were in addition fitted with accelerometers (HOBO Pendant G, Onset Computer Corporation, Bourne, MA, USA) secured to a distal forelimb with elastic wrap to monitor movement and orientation.

Sows were transferred between the transport and rendering trailer using a single file ramp. Flooring within the rendering trailer was bedded with wood shavings for the initial day of the trial at a level thought to be sufficient (~2.5 cm depth) and was increased to ~5 cm depth in days 2 and 3 to improve animal traction and reduce slips. Immediately upon completion of the loading process, foaming was initiated through the open canopy section at the top of the trailer. No top-ups of foam were needed, and sows were unable to be re-exposed to air once foaming was complete as the foam retained its level throughout the study period. No foam breakdown from convulsive activity was detected. Three individual foaming systems were used in tandem and consistently in all replicates. Personnel involved in the operation were wearing personal protective equipment including hats, gloves, glasses, boots and appropriate clothing. One system consisted of the equipment previously described for the small-scale study experiments where AF was produced using the CAFS and Spumifer nozzle. Two additional systems were each comprised of a gasoline-powered trash pump (Wacker Neuson, Menomonee Falls, WI, USA), with a 5.08-cm diameter suction hose connected to pump inlet and placed in a water reservoir containing 1% foam-water solution, a 3.8-cm diameter rubber firehose and a medium-expansion foam nozzle (Spumifer) connected to pump outlet via 5.08-cm diameter brass female National Pipe Straight Hose Thread (NPSH) and a 3.81 cm diameter male national hose (NH) fire hose adapter (W.W Grainger Inc, Lake Forest, IL, USA). Three staff members were placed on the roof of the rendering trailer, and each was responsible for operation of one medium expansion nozzle during the process. A fourth staff member was responsible for initiating the three foam systems and confirming operating status during the foam application process. Foam was applied until it reached the top of the trailer and overflowed. Following filling, the trailer was not disturbed for 15 min, after which the foam was evacuated using a leaf blower and death of each sow was confirmed based on lack of respirations, corneal reflex and body movement.

### 2.5. Data Management and Analysis

Accelerometry and swine body positional data retrieved from the HOBO pendant G devices were used to assign time to cessation of movement (COM) post the start of filling the depopulation space with gas or foam. Post-hoc visual confirmation of device stasis (no movement of *X*-, *Y*- or *Z*-axis) was used as an indication of COM and an unconscious state in individual sows. Electrocardiogram (ECG) tracings recovered from superficial cutaneous (Cardea Solo, small-scale trial) and subcutaneous (Star-Oddi DST-Centi-HRT, field trial) devices were reviewed to determine the time of death as indicated by asystole or presence of a fatal arrhythmia (3rd degree AV block, atrial standstill or ventricular fibrillation). Persistent or pulseless electrical activity (PEA) or electromechanical dissociation was defined as presence of any rhythm at the time of physical confirmation of death (i.e., clinical asystole).

Descriptive statistics including estimation of mean, median, standard deviation (SD), minimum, maximum and proportions were used to describe continuous and categorical variables of interest in the study, including time (s) to reach 5% oxygen concentration for gas-based trials (CO_2_ and N_2_), time to fill the dumpster (s) for foam-based trials (AF, CAF, and CAF-N_2_), time to cessation of movement for both gas- and foam-based trials and presence of foam at or beyond the tracheal bifurcation for foam trials. A Kaplan–Meier curve was used to visually describe time (s) to cessation of movement.

Two separate multivariable linear regression models were created to investigate the effect of treatment (CO_2_, N_2_, AF, CAF, and CAF-N_2_) on time to reach 5% oxygen (gas trials) and time to fill (for foam trials), while accounting for sow conscious state (anesthetized or conscious) as fixed effects. These analyses were conducted at the trial level. The Bonferroni correction was applied to adjust for multiple comparisons during contrasts. Linear model assumptions including homoscedasticity and normality of residuals were assessed by the Cook-Weisberg test for the former and the Shapiro–Wilk’s test for the latter.

In order to investigate the association between treatment and time to cessation of movement, a separate multivariable linear regression model was created. Because this analysis was conducted at the individual animal level, trial replicate (1 and 2) was added to the model as a fixed effect to account for the clustering of animals within a replicate. Adjustment for multiple comparisons and assessment of linear regression model assumptions were conducted as previously described.

Lastly, the association between the presence of foam at or beyond the tracheal bifurcation and depopulation foam treatment (CAF, CAF-N_2_ and AF) was investigated using a multivariable logistic regression model approach. Trial replicate (1 and 2) and animal conscious state (conscious or anesthetized) were included in the model as fixed effects. The Hosmer–Lemeshow test was used as a goodness-of-fit test.

All statistical analyses were conducted using Stata v.14 (StataCorp, College Station, TX, USA), and statistical significance was declared at *p* ≤ 0.05.

## 3. Results

### 3.1. Small-Scale Study Experiments

The mean time to reach 5% oxygen concentration inside the chamber during CO_2_ gas displacement was 88.3 s (SD: 7.41 s, range: 79.50–100.00 s and median: 89.0 s). The mean time to reach 5% oxygen concentration inside the chamber during N_2_ gas displacement was 101.5 s (SD: 30.90 s, range: 61.9–194 s, median: 74 s). A discrepancy between N_2_ time to 5% oxygen was observed within replicates applied to anesthetized sows, whereby replicate 1 took more than twice as long (194 s vs. 76 s) to reach 5% oxygen compared to replicate 2 (Table 1). No other discrepancy was found for the remaining anesthetized or conscious sow replicates.

The time to 5% oxygen in CO_2_ trials was significantly greater than in N_2_ trials, with the N_2_ requiring on average a 17-s decrease in time to fill when compared to CO_2_ (*p* = 0.04). No difference (*p* = 0.72) was observed when comparing the conscious and unconscious sow state in CO_2_ analyses. Neither the Cook–Weisberg (*p* = 0.39) nor the Shapiro–Wilk’s (*p* = 0.37) tests were statistically significant, indicating that the model assumptions for heteroskedasticity and normal distribution were met.

The mean time to fill the chamber was 154.75 s (SD: 33.92 s, range: 105–181 s and median: 166.50 s) for CAF trials, 183 s (SD: 27.2 s, range: 162–220 and median: 175 s) for CAF-N_2_ and 52.8 s (SD: 12.06 s, range: 35–62 s and median: 57 s) for AF. The fill time was different (*p* < 0.001) between the foam-based methods. A Bonferroni multiple comparisons test showed that both the CAF and CAF-N_2_ fill times were significantly longer compared to AF (CAF vs. AF, *p* = 0.001; CAF-N_2_ vs. AF, *p* < 0.001), while the CAF-N_2_ and CAF fill times were not different (*p* = 0.47). The sow state (conscious or anesthetized) was not significant in the model. Neither the Cook–Weisberg (*p* = 0.86) nor the Shapiro–Wilk’s (*p* = 0.99) tests were statistically significant, indicating that the model assumptions for heteroskedasticity and normal distribution were met.

Death was confirmed in 12/12 sows in the CO_2_ group (six anesthetized and six conscious) and 12/12 sows in the N_2_ group (six anesthetized and six conscious) following the maintenance of oxygen levels below 5% for 7 min. Death was confirmed in 12/12 sows in the CAF group (six anesthetized and six conscious), 12/12 sows in the CAF-N_2_ group (six anesthetized and six conscious) and 12/12 sows in the AF group (six anesthetized and six conscious) at 15 min following the completion of the filling process.

**Figure 1:**
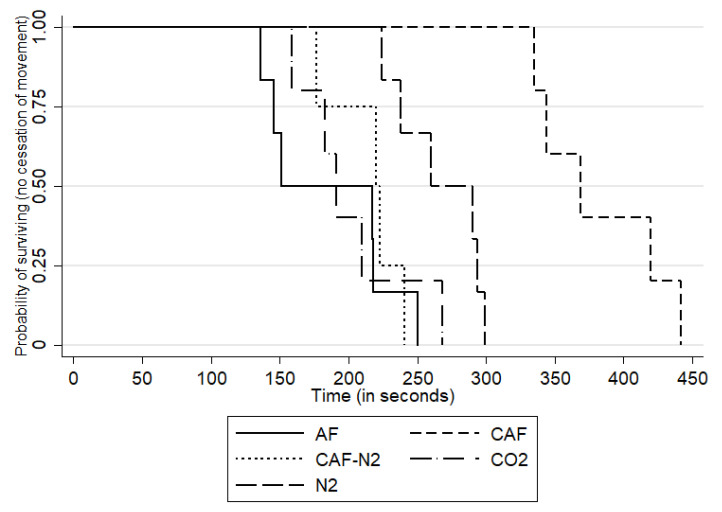
Kaplan–Meier curve describing within-group time to cessation of movement (COM) for foam- and gas-based methods aspirated foam (AF), compressed air foam (CAF), compressed nitrogen foam (CAF-N_2_), carbon dioxide gas (CO_2_) and nitrogen gas (N_2_) for two replicates of 3 pigs for each treatment. Attention should be paid to the 0.50 probability of surviving, which represents the time (in seconds) at which 50% of the animals in that treatment group had ceased movements (e.g., within approximately 150 s, animals within the AF group had ceased movement).

**Figure 2:**
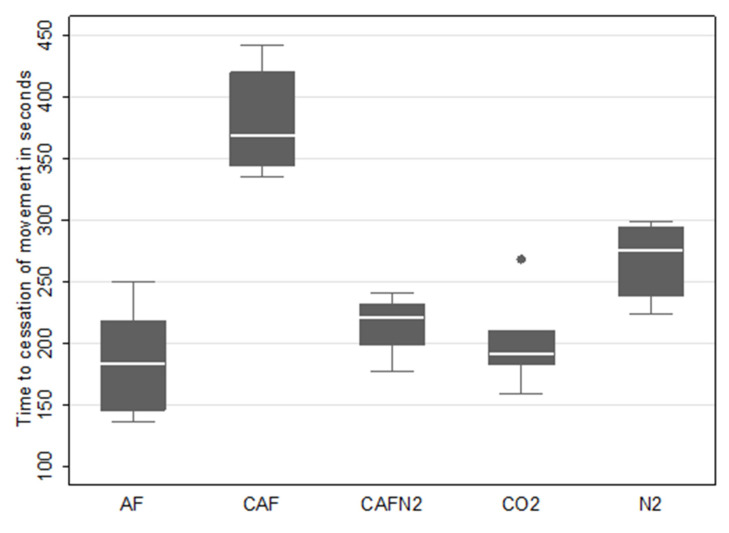
Box plots for time to cessation of movement (COM) for aspirated foam (AF), compressed air foam (CAF), compressed nitrogen foam (CAF-N_2_), carbon dioxide gas (CO_2_) and nitrogen gas (N_2_) for two replicates of 3 pigs for each treatment. Time to cessation of movement is presented in seconds and started upon treatment application.

For conscious animals, the mean time to COM during the CO_2_ gas trials was 202 s (SD: 41.10 s, median: 191 s and range: 159–268 s), while the mean time to COM during the N_2_ gas trials was 267.50 s (SD: 31.70 s, median: 275 s and range: 224–299 s). Within the foam application treatments to conscious sows, the mean time to COM was 382.00 s (SD: 47.10 s, range: 335–442 s and median: 369 s) for CAF, 215.20 s (SD: 27.1 s, range: 177–241 s and median: 221.50 s) for CAF-N_2_ and 186 s (SD: 47.8 s, range: 136–250 s and median: 184 s) for AF. The survival curves by treatment groups are shown in Figure 1 and the boxplots are shown in Figure 2.

The results from the multivariable linear model indicated an association between the treatment and time to cessation of movement, after accounting for replicates. The results from the multivariable model showed that CAF, CAFN_2_, CO_2_ and N_2_ methods were all associated with an increase in the time to cessation of movement when compared to the AF method, with all but the CO2 comparison being statistically significant. The magnitudes of increase in the time to COM for CAF, CAFN_2_, CO_2_ and N_2_ were of 201 s (*p* < 0.01), 42.50 s (*p* = 0.03), 10.4 s (*p* = 0.55) and 81.2 s (*p* < 0.01), respectively, relative to AF. The replicate was also statistically significant in this model, with the second replicate being associated with a reduction (s) of COM by 54.2 s as compared to the first replicate (*p* < 0.01). Results from the Bonferroni multiple comparisons tests across all treatments are shown in Table 2. Of note, the contrast between the CO2 and N_2_ treatments was statistically significant, with the N_2_ treatment being associated with an increase in time to COM of 70.7 s when compared to CO_2_ (*p* < 0.01). Lastly, neither the Cook–Weisberg (*p* = 0.33) nor the Shapiro–Wilk’s (*p* = 0.80) tests was statistically significant, showing that model assumptions were appropriately met.

A postmortem examination of the lower respiratory tract detected the presence of foam at or beyond the level of the tracheal bifurcation for 77.78% (28/36) of sows depopulated with foam-based methods: 91.7% (11/12) of CAF-depopulated sows, 58.3% (7/12) of CAF-N_2_-depopulated sows and 83.3% (10/12) of AF-depopulated sows. There was no significant difference in the proportion of animals with foam present (i.e., filling the airway) at or beyond the tracheal bifurcation among the different foam-based methods (*p* = 0.13). No difference in the odds of having foam at or beyond the tracheal bifurcation was found (OR = 0.16 (95% CI: 0.02–1.33), *p* = 0.09) between replicates. Additionally, no difference between anesthetized and conscious animals was found (OR = 6.11(95% CI: 0.75–49.65) *p* = 0.09) of having foam at or beyond the bifurcation when compared to anesthetized animals. The model fit well according to the Hosmer–Lemeshow test (*p* = 0.63).

Tracings for ECG were recovered from 41 of the 60 external CardeaSolo devices placed on individual sows and independently examined (Table 3). Data recovery was negatively impacted in conscious sow groups due to several factors, including increased technical difficulty achieving adequate lead contact with the skin during the placement. Devices were also more likely to be dislodged during animal–animal contact in conscious groups. Fatal arrhythmias, including third-degree atrioventricular block, atrial standstill and ventricular fibrillation were noted in 53.6% (15/28) anesthetized sows and 69.2% (9/13) of conscious sows (Table 3). The presence of PEA was noted in 15 anesthetized sows and five conscious sows; this was independent of the presence of fatal arrhythmia and the fact that death had been confirmed clinically at the time that PEA was occurring.

### 3.2. Field Trial: Depopulation in Modified Rendering Trailer with Aspirated Foam (AF)

The first experimental group of sows (group 1) was divided into two subsets of 25 (group 1A) and 20 sows (group 1B), with each loaded and depopulated separately. The time to load was approximately 19 min and 8 min for the two subgroups, respectively, providing an aggregate loading time of 27 min. A modification of the loading strategy, involving the addition of gating in the middle of the trailer and the inclusion of an additional depth of wood shavings, improved the loading time in subsequent experimental groups two and three, which were loaded as single cohorts. The time to load was approximately 17 min for both groups two (n = 44) and three (n = 45). Overall, the mean time to load was 20 min per group of 44–45 sows, corresponding with a loading time per individual sow of approximately 27 s. The mean time to fill during the four applications of AF in the depopulation trailer was 103.8 s (SD: 5.0 s, range: 92–115 s and median: 104 s).

**Figure 3:**
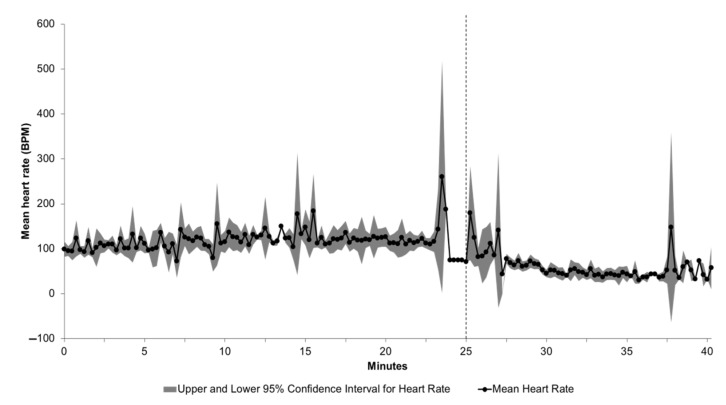
Mean heart rate in beats per minute (BPM) among sows fitted with Star-Oddi devices in groups 1A, 1B, 2 and 3. Note: only HR values with the highest quality rating (QI = 0) are represented in these data (n = 1–11 for each time point). *X*-axis shows relative time across the multiple groups; data shown cover 25 min prior to and 15 min following fill start time (denoted by the vertical dashed line). Heart rate data in the subcutaneous devices was collected using ECG-based proprietary technology and algorithms (Star-Oddi). Readers should note that HR data indicate only electrical activity and not cardiac function; persistent electrical activity (PEA) may be detected following confirmation of clinical asystole by physical methods (e.g., auscultation or pulse detection).

The mean time to COM (based on data collected from HOBO devices and inclusive of foam filling time) across the four applications was 128.0 s (SD: 18.6 s, range: 98–162 s and median: 130.5 s). The mean time to COM within the four applications was: 136.0 s in group 1A (SD: 23.3 s, range: 108–162 s and median: 137.0 s), 142.7 s in group 1B (SD: 9.1 s, range 134–155 s and median: 141.0 s), 109 s in group 2 (SD: 9.4 s, range: 98–120 s and median: 109.5 s), and 124 s in group 3 (SD: 12.2 s, range: 108–134 s and median: 127.0 s). An estimated 1136 L of 1% foam–water solution was required to completely fill the 68,000 L (68 m^3^) volume-rendering trailer at each application, indicating an approximate expansion ratio of 45:1, which is typical for medium expansion foam. Death was confirmed in all sows by the lack of movement at 15 min following the completion of the filling process. The results from mean heart rate estimates (25 min prior to and 15 min following fill start time) are displayed in Figure 3.

## 4. Discussion

The small-scale trials designed to determine the efficacy of carbon dioxide gas, nitrogen gas, compressed air foam, CAF made with nitrogen gas and aspirated foam methodologies for depopulating sows were all deemed successful. Each of the foam and gas combinations induced COM well within the allocated time period of 15 min and the 7-min saturation time post-completed chamber fill and 5% O_2_ concentration level. Among the gas-only methods, N_2_ had the longest mean time to COM at 267.5 s (4 min 28 s), while CO_2_ had the shortest at 202.2 s (3 min 22 s). Water-based aspirated foam (AF) had the shortest overall time to COM at 186.3 s (3 min 6 s), followed by CAF-N_2_ at 215.2 s (3 min 35 s), while CAF had the longest at 382 s (6 min, 22 s). The N_2_ results should be interpreted in the light of a longer fill time during the anesthetized sow trial, which indicates there may be practical issues for N_2_ delivery in the field. The observed difference between replicates for N_2_ in anesthetized sows is difficult to ascertain as it was only observed once for one replicate and treatment but may have been influenced by environmental factors such as ambient temperature and humidity, a factor to consider and account for in future field studies or implementation during different weather conditions. The relative time to COM between gas and foam methodologies for this study is comparable to findings using the same gas and foam treatments in layers, with the exception of AF, which showed the slowest latency to COM [23,24,25]. Interestingly, as discussed in Gurung et al. [23], it is possible that the air-filled foam bubbles may prolong the time to death in the layers used in their study. In contrast, despite the use of identical foam proportioning units in both studies, our study found that AF had both the fastest fill time and shortest latency to COM and was comparable to using CO_2_. It is possible that the difference in the foam water solution ratio (3.5% vs. 1–2%) may have impacted the oxygen levels in the foam bubbles, allowing for a lower foam oxygen content in the foam used in the present study [26]. Thus, a plausible explanation for the observed quicker time to COM of pigs when compared to poultry may be due to differences in body size and, in extension, oxygen needs and conservation in a hypoxic environment [27]. In addition, there is a possibility that the foam characteristics may have behaved differently between trials and replicates based on the gas used as any chemical or physical composition analysis of the bubbles was not conducted. The time to COM for our sows in the CO_2_ treatment was slightly lower when compared to previously reported findings regarding the latency to the last movement for weaning pigs of 225 to 450 s depending on chamber pre-fill or flow rate [26], but similar to earlier findings by Çavuşoğlu et al. reporting COM at 200 to 220 s post-exposure [27].

The ECG tracings were of sufficient quality for interpretation in 41 cases. Fatal arrhythmias, such as third-degree atrioventricular block, atrial standstill and ventricular fibrillation, were identified in 24/41 cases. The remaining 17 animals, as well as three of the animals with fatal rhythms, developed PEA. Acidosis is a known factor that exacerbates ventricular mechanical failure and impairs the cardiovascular system due to its negative inotropic and vasodilatory effects [28]. Together, the failing mechanical activity of the myocardium and vasodilation worsens the acidosis, which results in myocardial electromechanical dissociation, unconsciousness and death. It should be noted that the observed peaks in heart rate prior and shortly after the foam fill initiation shown in Figure 3 may be indicative of stress response and may be a welfare concern worth investigating more in detail.

For the large-scale study, the mean time to COM using AF was 128 s. To our knowledge, no comparable large-scale depopulation studies using aspirated foam in swine exist to date. Thus, based on the similar results derived from our AF and CO_2_ trials, the closest comparison would be other large-scale or field studies using CO_2_. Using that comparison, the time to COM in our study was similar to the time to loss of righting reflex that has been previously reported [29], but contrasting in comparison to the estimated 30 to 60 s and up to 4.5 min to COM reported using a 14.5 m^3^ trailer in two different field trials [15]. However, as the mentioned study used a smaller trailer by volume and a 62% CO_2_ atmosphere instead of the more commonly used 95% CO_2_, it is possible that the faster fill time due to the smaller trailer volume was offset by the lower CO_2_ concentration [15].

Despite the successful implementation of both gases and foam in the present small-scale trial and other published trials, the gas options evaluated may be difficult to carry out on a large scale despite developed prototypes [16,29,30]. Potential problems with gas-based methods include its sourcing, delivery and storage, vaporization rate and risk of operator exposure both to the gas itself and as a potential pressurized equipment malfunction [31,32,33]. In contrast, the results of the present studies indicate that water-based foam alone may be an acceptable alternative for large-scale depopulation in swine due to its relative ease of use, minimal risks to personnel, use of less volume when compared to inert gas implementation and the established use in poultry [23,33,34,35,36]. Without the addition of gas(es) to foam, depopulation sites need not be equipped with gas storage tanks or vaporizers. Furthermore, foaming equipment such as pumps, hoses, foam concentrate and water tanks are readily available from industrial and firefighting suppliers, and all materials can be stored without permits or specialty tanks. Coupled with foaming supplies already present within the USDA, National Veterinary Stockpile, this combination of factors and the lack of literature on foaming application for the swine species warranted the extension of only the AF protocol in our present work to the pilot field trial portion of the present study.

Results from the field trials support the finding that the depopulation of swine can be successfully achieved using water-based foaming. The findings indicate that the AF process could be successfully scaled up while maintaining a quick foam fill time for a significantly larger mobile depopulation unit. The mean fill time and COM time for the field trial were reduced by approximately 30% when compared to the small-scale trial, whereas the use of AF for the depopulation of swine resulted in a demonstrated promise for field application in the present study; therefore, important logistical aspects need to be considered for successful field application in an even larger scale, that of modern swine production settings. Our findings indicate that animal loading can be a bottleneck that reduces efficiency and must be addressed. In addition, the speed of the foam fill time is an essential factor in time to animal death; therefore, a focus on equipment size/capacity as well as training of personnel to operate the process will be necessary. Increasing the number of pumps and foam proportioning units may decrease fill time but may also require additional personnel, leading to increased costs in labor and equipment.

Moreover, the use of a mobile rendering trailer as a depopulation vessel in the field provides additional benefits and opportunities for decontamination, carcass disposal and worker safety as the activities are performed away from the production environment [17,37]. When animals walk into the depopulation trailer under their own power, the human labor required to carry out the depopulation event and remove carcasses from the site (if a dump trailer is used) is reduced. In addition, a watertight rendering trailer allows rapid foam application to a large group of animals and can be moved to multiple sites as needed. Of note, the decision process for animal movement under the presence of disease and the implications on welfare are inherent in all depopulation decisions; therefore, the choice of depopulation method(s) will include situation-dependent decisions in consultation with authorities.

The results of the present study clearly indicate that AF is a viable agent for the mass depopulation of mature swine in modified rendering trailers. Additional research is needed to validate foam in younger swine, to investigate the impact on animal welfare, physiological differences between drowning and depopulation by foam, and to assess the dwell time needed to ensure non-recovery. Furthermore, it is important to note that the sample size used here was modest; this was due to the lack of available information on the topic as well as ethical considerations given the nature of the research. We also had a lack of independence for the analysis conducted at the animal level, given that the animals were clustered within dumpster replicates/ trailers. Even though we attempted to build robust multivariable models to account for this issue, the reader should interpret our results with caution. Future research considerations include other commercially available foam concentrates, lower-quality trash pumps, varying stocking density in trailers, containing foam following application and success of foaming under extreme heat or cold conditions.

## 5. Conclusions

Our results show that AF resulted in the cessation of movement and efficacy to induce death during a dwell period of 15 min that was comparable to CO_2._ The ability to stockpile agents coupled with only requiring low-cost and readily available equipment makes AF a practical depopulation solution for swine. The initial large-scale trials using AF in a modified rendering trailer showed promising results as they allowed for multiple animals to be moved to a designated depopulation area and to be terminated simultaneously. The use of a modified rendering trailer also helps overcome some of the inherent hurdles of performing depopulation within the swine facility environment and eases carcass disposal. The preapproved use of AF in poultry depopulation by AVMA in combination with existing AF-generating units in the National Veterinary Stockpile, as well as the relative safety and ease of use, makes AF an attractive agent to use in local, regional or national depopulation emergencies.

## Figures and Tables

**Table 1:** Fill time in seconds (s) to reach 5% oxygen levels during a small-scale trial of gas-displacement for carbon dioxide (CO_2_) and nitrogen (N_2_) gas for anesthetized and conscious pigs for two replicates of 3 pigs for each treatment.

Replicate	CO_2_ Anesthetized	CO_2_ Conscious	N_2_ Anesthetized	N_2_ Conscious
1	96	86	194	72
2	79	92	76	64

**Table 2:** Reported model *p*-values with Bonferroni correction for pair-wise comparisons of time to cessation of movement (COM) for two replicates of 3 pigs for each treatment between foam- and gas-based methods; aspirated foam, AF (186.3 s); compressed air foam, CAF (382.0 s); compressed nitrogen foam, CAF-N_2_ (215.3 s); and carbon dioxide gas, CO_2_ (202.2 s) nitrogen gas, N_2_ (267.7 s). *p*-values considered significant at the level of 0.05.

	AF	CAF	CAF-N_2_	CO_2_
CAF	<0.001			
CAF-N_2_	<0.33	<0.001		
CO_2_	1.000	<0.001	1.000	
N_2_	<0.001	<0.001	0.50	0.006

**Table 3:** Frequency (in number; N) of persistent electrical activity (PEA) and fatal arrhythmias (3rd degree AV block, atrial standstill and ventricular fibrillation) identified in electrocardiograph (ECG) tracings recovered from individual swine. Treatments included: aspirated foam (AF), compressed air foam (CAF), compressed nitrogen foam (CAF-N_2_), carbon dioxide gas (CO_2_) and nitrogen gas (N_2_) applied to anesthetized and conscious swine.

			Fatal Arrhythmias		
	Number ECGs Readable/Number Animals Monitored	PEA	3rd Degree AV Block	Atrial Standstill	Ventricular Fibrillation
AF-Anesthetized	6/6	1	2	3	
AF-Conscious	3/6	1		2	
CAF-Anesthetized	6/6	3	3	1	1
CAF-Conscious	1/6		1		
CAF-N_2_-Anesthetized	6/6	3	2	1	
CAF-N_2_-Conscious	5/6	2	1	2	
CO_2_-Anesthetized	5/6	3	1		
CO_2_-Conscious	0/6				
N_2_-Anesthetized	5/6	5	1		
N_2_-Conscious	4/6	2	1	1	1
Total Anesthetized	28/30	15	9	5	1
Total Conscious	13/30	5	3	5	1

## Data Availability

Data available upon request.
